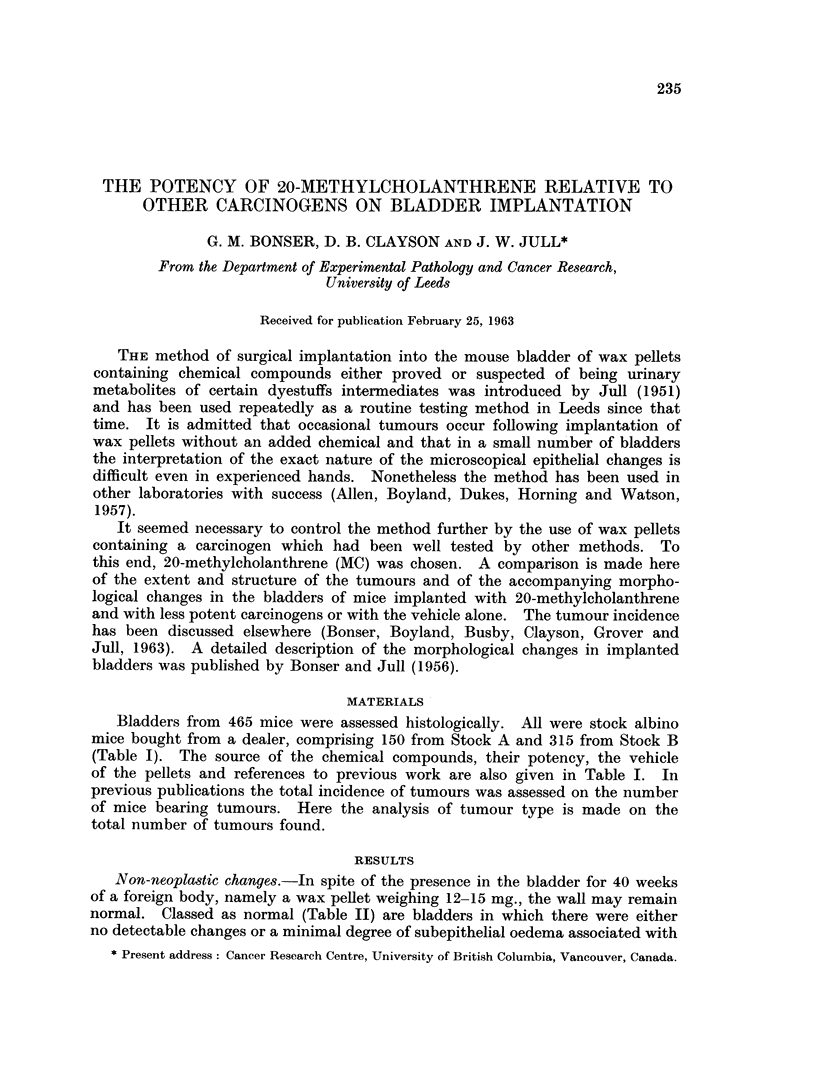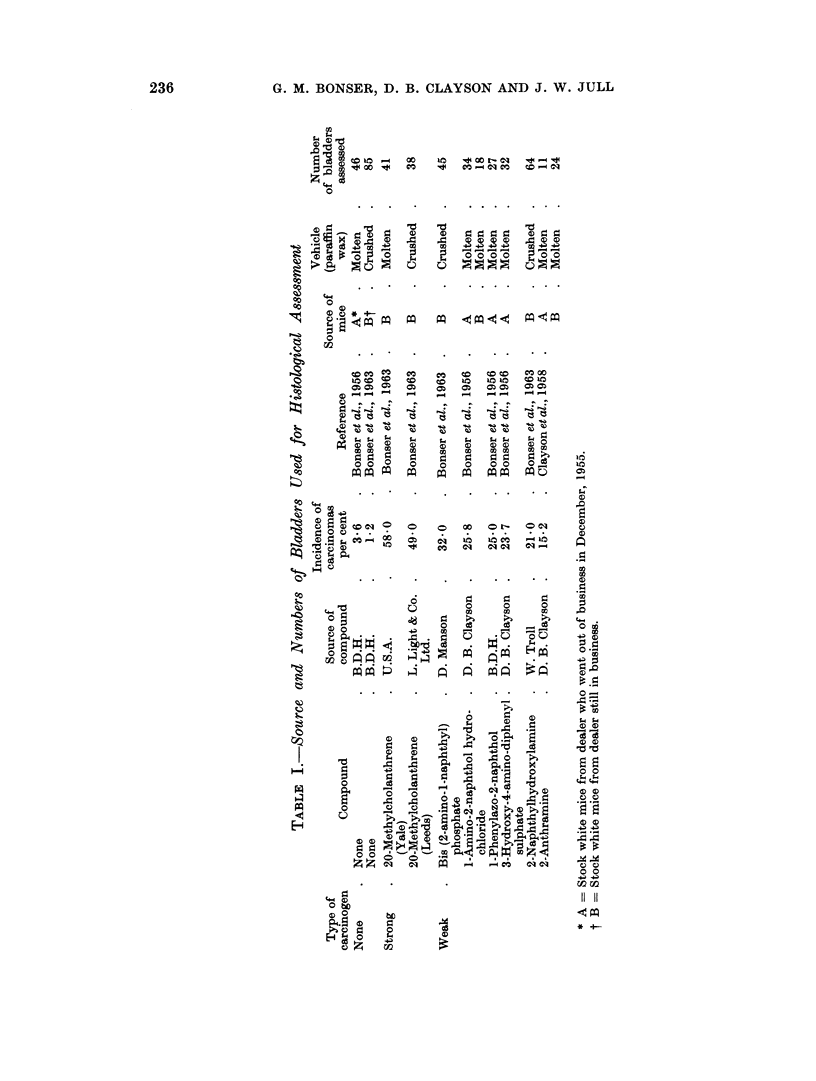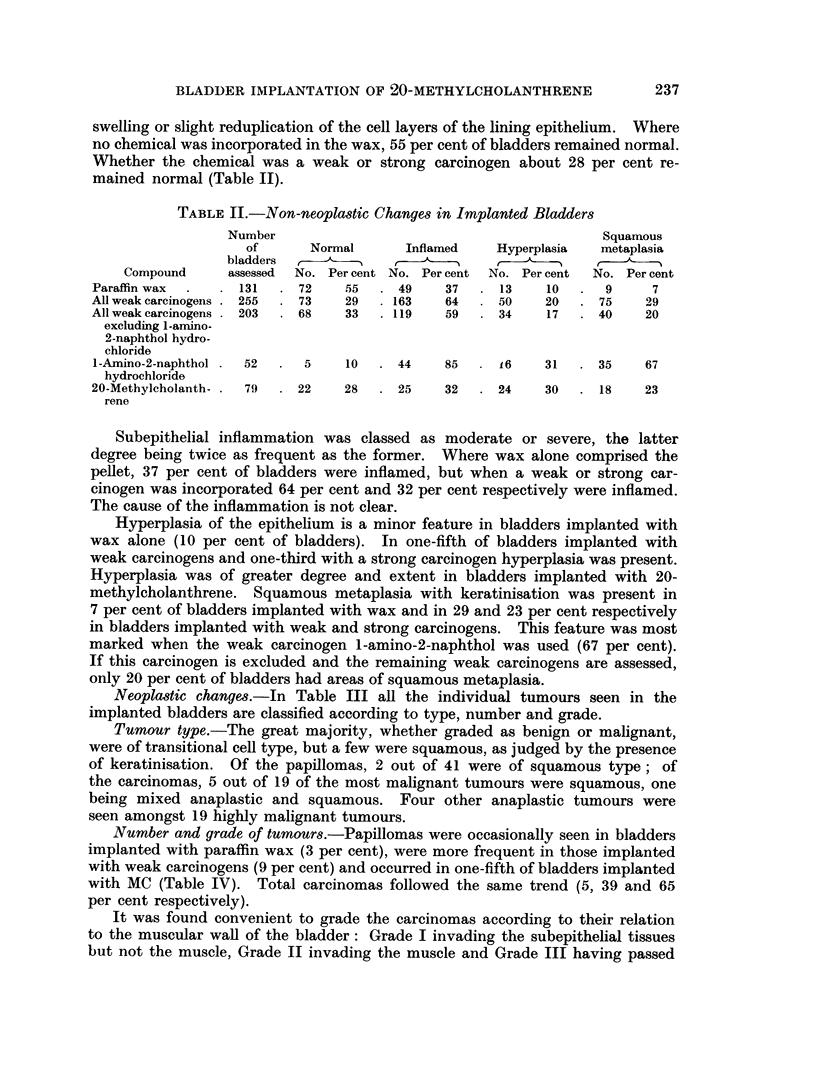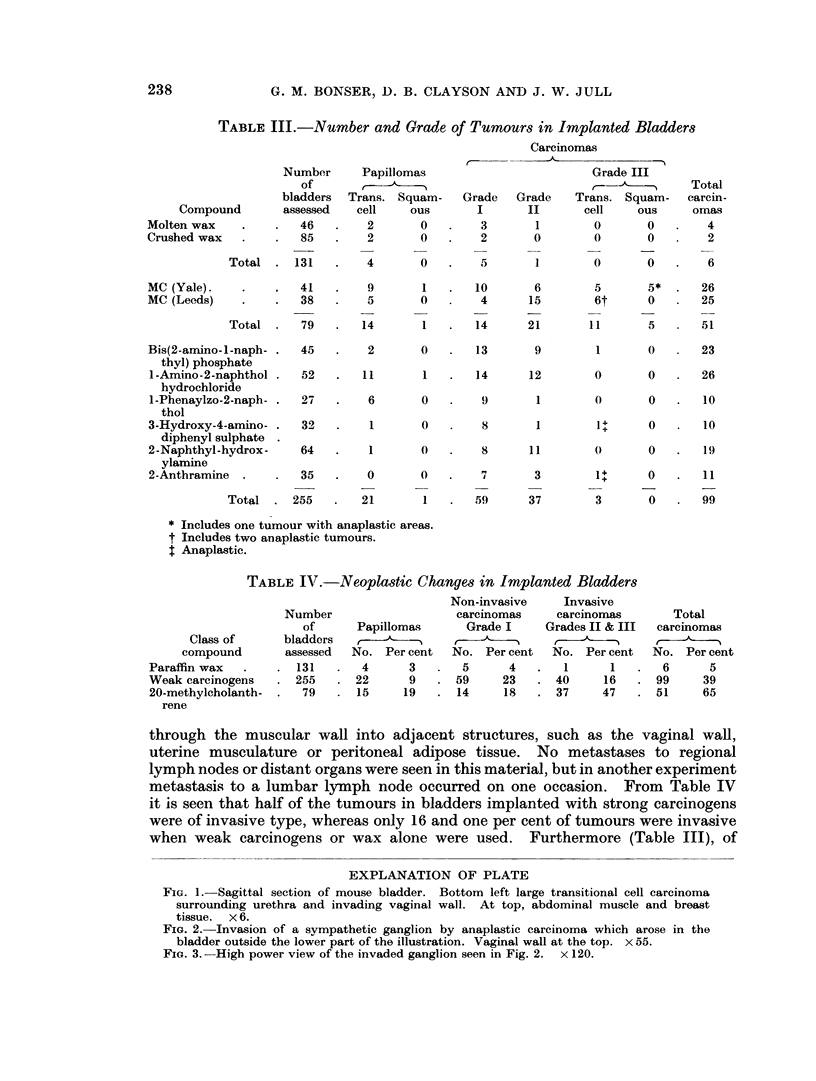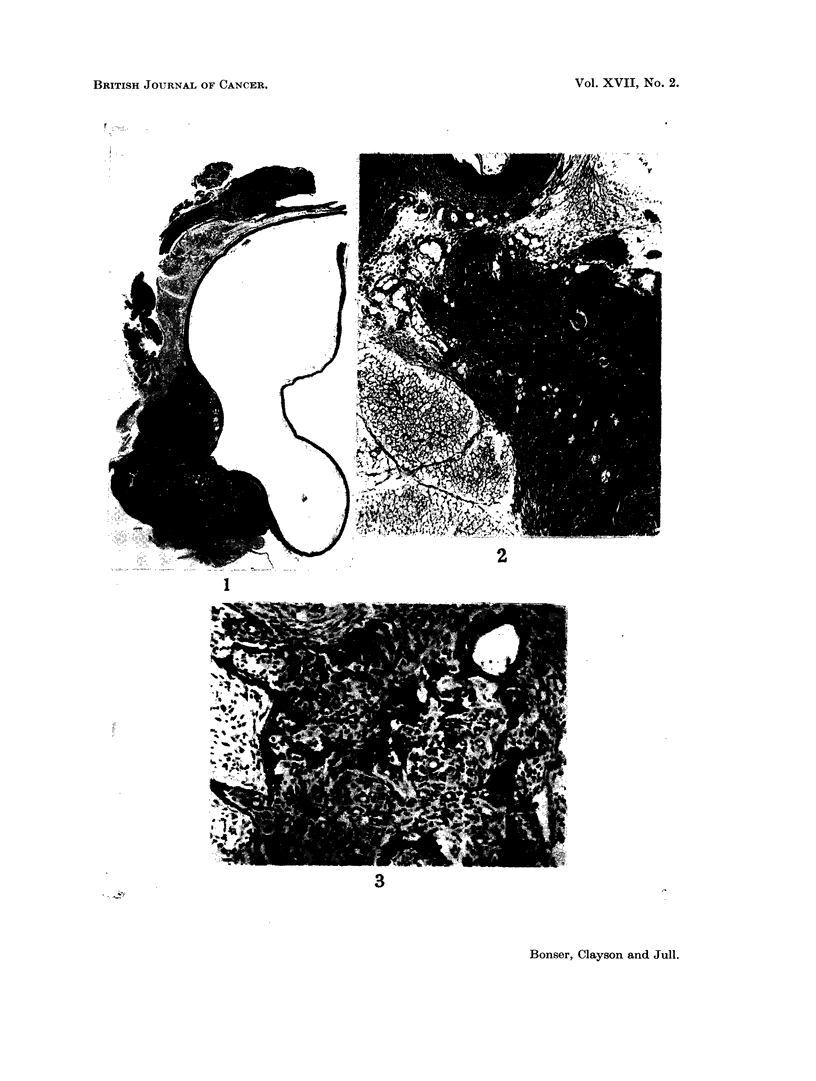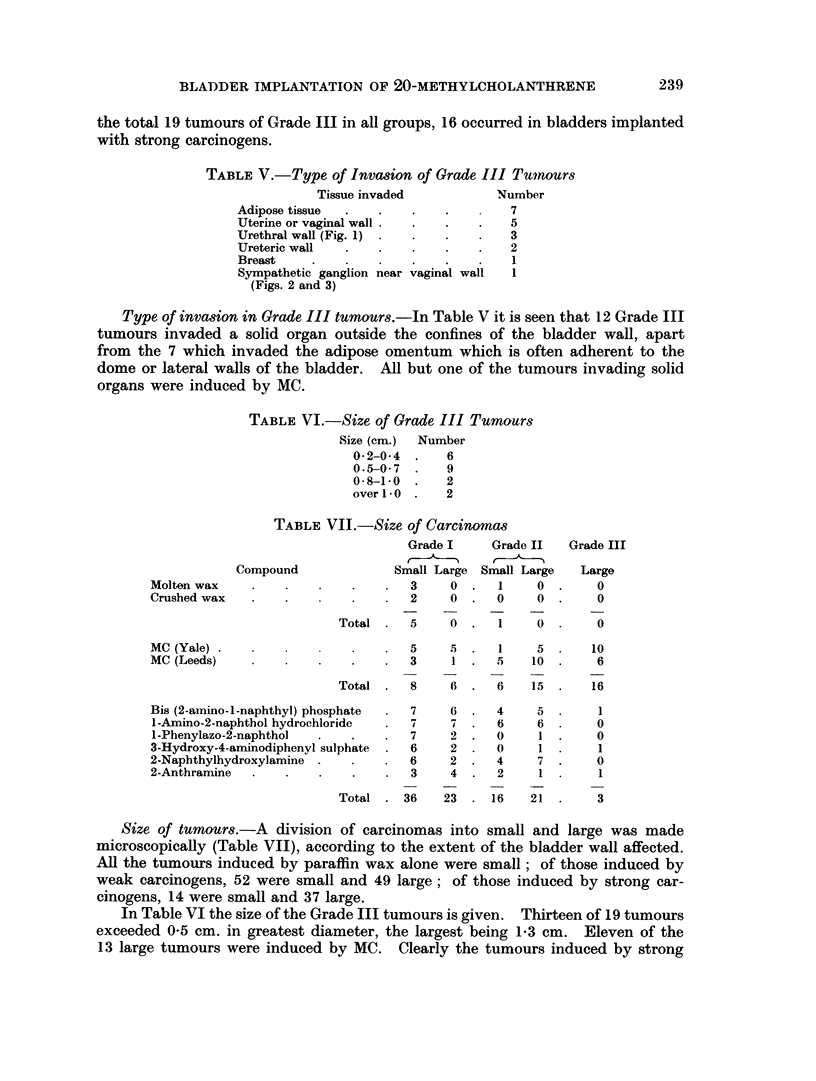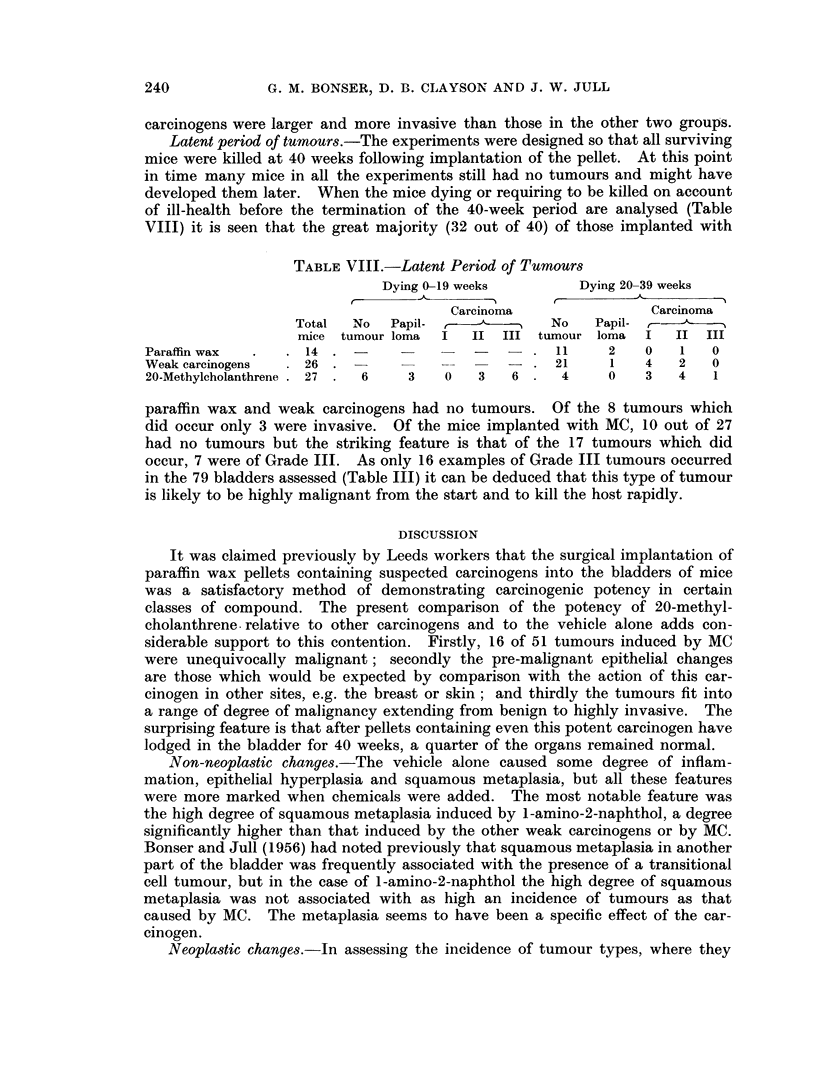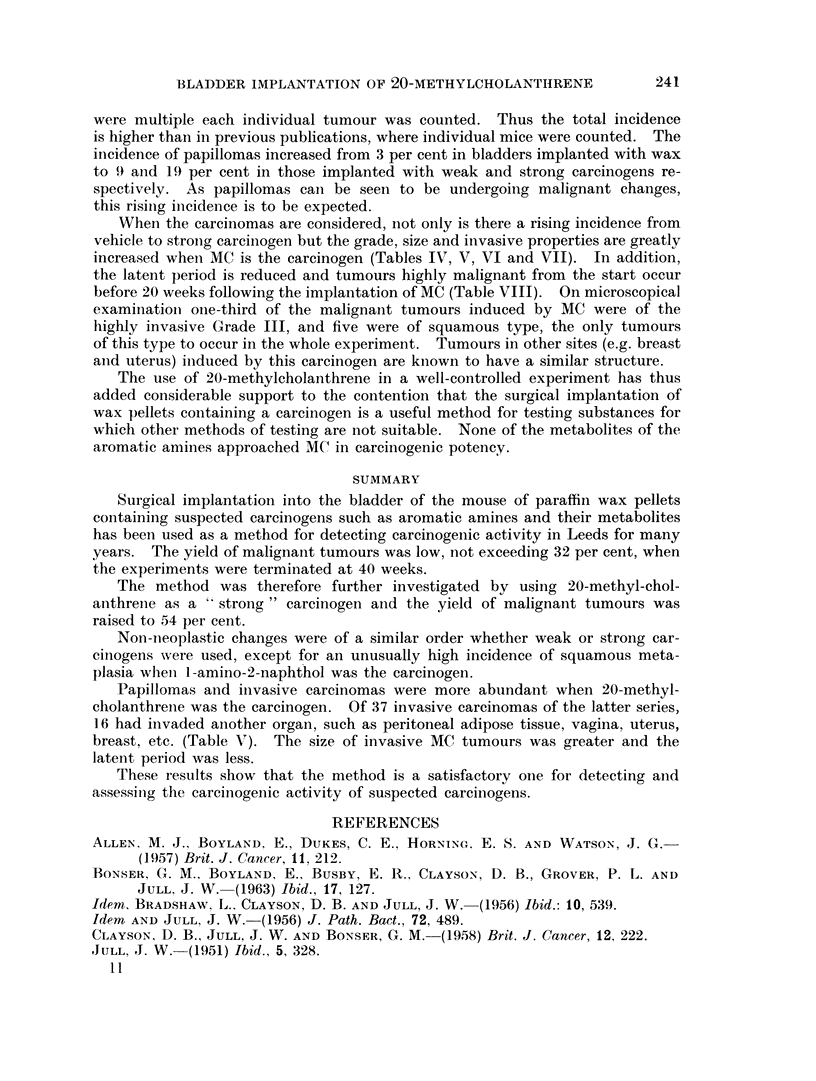# The Potency of 20-Methylcholanthrene Relative to Other Carcinogens on Bladder Implantation

**DOI:** 10.1038/bjc.1963.33

**Published:** 1963-06

**Authors:** G. M. Bonser, D. B. Clayson, J. W. Jull

## Abstract

**Images:**


### THE POTENCY OF 20-METHYLCHOLANTHRENE RELATIVE TO OTHER CARCINOGENS ON BLADDER IMPLANTATION

G. M. BONSER, D. B. CLAYSON AND J. W. JULL*

*From the Department of Experimental Pathology and Cancer Research, University of Leeds*

Received for publication February 25, 1963

THE method of surgical implantation into the mouse bladder of wax pellets containing chemical compounds either proved or suspected of being urinary metabolites of certain dyestuffs intermediates was introduced by Jull (1951) and has been used repeatedly as a routine testing method in Leeds since that time. It is admitted that occasional tumours occur following implantation of wax pellets without an added chemical and that in a small number of bladders the interpretation of the exact nature of the microscopical epithelial changes is difficult even in experienced hands. Nonetheless the method has been used in other laboratories with success (Allen, Boyland, Dukes, Horning and Watson, 1957).

It seemed necessary to control the method further by the use of wax pellets containing a carcinogen which had been well tested by other methods. To this end, 20-methylcholanthrene (MC) was chosen. A comparison is made here of the extent and structure of the tumours and of the accompanying morphological changes in the bladders of mice implanted with 20-methylcholanthrene and with less potent carcinogens or with the vehicle alone. The tumour incidence has been discussed elsewhere (Bonser, Boyland, Busby, Clayson, Grover and Jull, 1963). A detailed description of the morphological changes in implanted bladders was published by Bonser and Jull (1956).

### MATERIALS

Bladders from 465 mice were assessed histologically. All were stock albino mice bought from a dealer, comprising 150 from Stock A and 315 from Stock B (Table I). The source of the chemical compounds, their potency, the vehicle of the pellets and references to previous work are also given in Table I. In previous publications the total incidence of tumours was assessed on the number of mice bearing tumours. Here the analysis of tumour type is made on the total number of tumours found.

### RESULTS

*Non-neoplastic changes.*—In spite of the presence in the bladder for 40 weeks of a foreign body, namely a wax pellet weighing 12–15 mg., the wall may remain normal. Classed as normal (Table II) are bladders in which there were either no detectable changes or a minimal degree of subepithelial oedema associated with

* Present address : Cancer Research Centre, University of British Columbia, Vancouver, Canada.

TABLE I.—*Source and Numbers of Bladders Used for Histological Assessment*

| Type of carcinogen | Compound | Source of compound | Incidence of carcinomas per cent | Reference | Source of mice | Vehicle (paraffin wax) | Number of bladders assessed |
|---|---|---|---|---|---|---|---|
| None | None | B.D.H. | 3·6 | Bonser *et al.*, 1956 | A* | Molten | 46 |
| | None | B.D.H. | 1·2 | Bonser *et al.*, 1963 | B† | Crushed | 85 |
| Strong | 20-Methylcholanthrene (Yale) | U.S.A. | 58·0 | Bonser *et al.*, 1963 | B | Molten | 41 |
| | 20-Methylcholanthrene (Leeds) | L. Light & Co. Ltd. | 49·0 | Bonser *et al.*, 1963 | B | Crushed | 38 |
| Weak | Bis (2-amino-1-naphthyl) phosphate | D. Manson | 32·0 | Bonser *et al.*, 1963 | B | Crushed | 45 |
| | 1-Amino-2-naphthol hydrochloride | D. B. Clayson | 25·8 | Bonser *et al.*, 1956 | A | Molten | 34 |
| | | | | | B | Molten | 18 |
| | 1-Phenylazo-2-naphthol | B.D.H. | 25·0 | Bonser *et al.*, 1956 | A | Molten | 27 |
| | 3-Hydroxy-4-amino-diphenyl sulphate | D. B. Clayson | 23·7 | Bonser *et al.*, 1956 | A | Molten | 32 |
| | 2-Naphthylhydroxylamine | W. Troll | 21·0 | Bonser *et al.*, 1963 | B | Crushed | 64 |
| | 2-Anthramine | D. B. Clayson | 15·2 | Clayson *et al.*, 1958 | A | Molten | 11 |
| | | | | | B | Molten | 24 |

* A = Stock white mice from dealer who went out of business in December, 1955.
† B = Stock white mice from dealer still in business.

swelling or slight reduplication of the cell layers of the lining epithelium. Where no chemical was incorporated in the wax, 55 per cent of bladders remained normal. Whether the chemical was a weak or strong carcinogen about 28 per cent remained normal (Table II).

TABLE II.—*Non-neoplastic Changes in Implanted Bladders*

| Compound | Number of bladders assessed | Normal No. | Normal Per cent | Inflamed No. | Inflamed Per cent | Hyperplasia No. | Hyperplasia Per cent | Squamous metaplasia No. | Squamous metaplasia Per cent |
|---|---|---|---|---|---|---|---|---|---|
| Paraffin wax | 131 | 72 | 55 | 49 | 37 | 13 | 10 | 9 | 7 |
| All weak carcinogens | 255 | 73 | 29 | 163 | 64 | 50 | 20 | 75 | 29 |
| All weak carcinogens excluding 1-amino-2-naphthol hydrochloride | 203 | 68 | 33 | 119 | 59 | 34 | 17 | 40 | 20 |
| 1-Amino-2-naphthol hydrochloride | 52 | 5 | 10 | 44 | 85 | 16 | 31 | 35 | 67 |
| 20-Methylcholanthrene | 79 | 22 | 28 | 25 | 32 | 24 | 30 | 18 | 23 |

Subepithelial inflammation was classed as moderate or severe, the latter degree being twice as frequent as the former. Where wax alone comprised the pellet, 37 per cent of bladders were inflamed, but when a weak or strong carcinogen was incorporated 64 per cent and 32 per cent respectively were inflamed. The cause of the inflammation is not clear.

Hyperplasia of the epithelium is a minor feature in bladders implanted with wax alone (10 per cent of bladders). In one-fifth of bladders implanted with weak carcinogens and one-third with a strong carcinogen hyperplasia was present. Hyperplasia was of greater degree and extent in bladders implanted with 20-methylcholanthrene. Squamous metaplasia with keratinisation was present in 7 per cent of bladders implanted with wax and in 29 and 23 per cent respectively in bladders implanted with weak and strong carcinogens. This feature was most marked when the weak carcinogen 1-amino-2-naphthol was used (67 per cent). If this carcinogen is excluded and the remaining weak carcinogens are assessed, only 20 per cent of bladders had areas of squamous metaplasia.

*Neoplastic changes.*—In Table III all the individual tumours seen in the implanted bladders are classified according to type, number and grade.

*Tumour type.*—The great majority, whether graded as benign or malignant, were of transitional cell type, but a few were squamous, as judged by the presence of keratinisation. Of the papillomas, 2 out of 41 were of squamous type ; of the carcinomas, 5 out of 19 of the most malignant tumours were squamous, one being mixed anaplastic and squamous. Four other anaplastic tumours were seen amongst 19 highly malignant tumours.

*Number and grade of tumours.*—Papillomas were occasionally seen in bladders implanted with paraffin wax (3 per cent), were more frequent in those implanted with weak carcinogens (9 per cent) and occurred in one-fifth of bladders implanted with MC (Table IV). Total carcinomas followed the same trend (5, 39 and 65 per cent respectively).

It was found convenient to grade the carcinomas according to their relation to the muscular wall of the bladder : Grade I invading the subepithelial tissues but not the muscle, Grade II invading the muscle and Grade III having passed

TABLE III.—*Number and Grade of Tumours in Implanted Bladders*

| Compound | Number of bladders assessed | Papillomas Trans. cell | Papillomas Squamous | Carcinomas Grade I | Carcinomas Grade II | Carcinomas Grade III Trans. cell | Carcinomas Grade III Squamous | Total carcinomas |
|---|---|---|---|---|---|---|---|---|
| Molten wax | 46 | 2 | 0 | 3 | 1 | 0 | 0 | 4 |
| Crushed wax | 85 | 2 | 0 | 2 | 0 | 0 | 0 | 2 |
| Total | 131 | 4 | 0 | 5 | 1 | 0 | 0 | 6 |
| MC (Yale) | 41 | 9 | 1 | 10 | 6 | 5 | 5* | 26 |
| MC (Leeds) | 38 | 5 | 0 | 4 | 15 | 6† | 0 | 25 |
| Total | 79 | 14 | 1 | 14 | 21 | 11 | 5 | 51 |
| Bis(2-amino-1-naphthyl) phosphate | 45 | 2 | 0 | 13 | 9 | 1 | 0 | 23 |
| 1-Amino-2-naphthol hydrochloride | 52 | 11 | 1 | 14 | 12 | 0 | 0 | 26 |
| 1-Phenaylzo-2-naphthol | 27 | 6 | 0 | 9 | 1 | 0 | 0 | 10 |
| 3-Hydroxy-4-amino-diphenyl sulphate | 32 | 1 | 0 | 8 | 1 | 1‡ | 0 | 10 |
| 2-Naphthyl-hydroxylamine | 64 | 1 | 0 | 8 | 11 | 0 | 0 | 19 |
| 2-Anthramine | 35 | 0 | 0 | 7 | 3 | 1‡ | 0 | 11 |
| Total | 255 | 21 | 1 | 59 | 37 | 3 | 0 | 99 |

* Includes one tumour with anaplastic areas.
† Includes two anaplastic tumours.
‡ Anaplastic.

TABLE IV.—*Neoplastic Changes in Implanted Bladders*

| Class of compound | Number of bladders assessed | Papillomas No. | Papillomas Per cent | Non-invasive carcinomas Grade I No. | Non-invasive carcinomas Grade I Per cent | Invasive carcinomas Grades II & III No. | Invasive carcinomas Grades II & III Per cent | Total carcinomas No. | Total carcinomas Per cent |
|---|---|---|---|---|---|---|---|---|---|
| Paraffin wax | 131 | 4 | 3 | 5 | 4 | 1 | 1 | 6 | 5 |
| Weak carcinogens | 255 | 22 | 9 | 59 | 23 | 40 | 16 | 99 | 39 |
| 20-methylcholanthrene | 79 | 15 | 19 | 14 | 18 | 37 | 47 | 51 | 65 |

through the muscular wall into adjacent structures, such as the vaginal wall, uterine musculature or peritoneal adipose tissue. No metastases to regional lymph nodes or distant organs were seen in this material, but in another experiment metastasis to a lumbar lymph node occurred on one occasion. From Table IV it is seen that half of the tumours in bladders implanted with strong carcinogens were of invasive type, whereas only 16 and one per cent of tumours were invasive when weak carcinogens or wax alone were used. Furthermore (Table III), of

### EXPLANATION OF PLATE

FIG. 1.—Sagittal section of mouse bladder. Bottom left large transitional cell carcinoma surrounding urethra and invading vaginal wall. At top, abdominal muscle and breast tissue. ×6.

FIG. 2.—Invasion of a sympathetic ganglion by anaplastic carcinoma which arose in the bladder outside the lower part of the illustration. Vaginal wall at the top. ×55.

FIG. 3.—High power view of the invaded ganglion seen in Fig. 2. ×120.

1

2

3

the total 19 tumours of Grade III in all groups, 16 occurred in bladders implanted with strong carcinogens.

TABLE V.—*Type of Invasion of Grade III Tumours*

| Tissue invaded | Number |
|---|---|
| Adipose tissue | 7 |
| Uterine or vaginal wall | 5 |
| Urethral wall (Fig. 1) | 3 |
| Ureteric wall | 2 |
| Breast | 1 |
| Sympathetic ganglion near vaginal wall (Figs. 2 and 3) | 1 |

*Type of invasion in Grade III tumours.*—In Table V it is seen that 12 Grade III tumours invaded a solid organ outside the confines of the bladder wall, apart from the 7 which invaded the adipose omentum which is often adherent to the dome or lateral walls of the bladder. All but one of the tumours invading solid organs were induced by MC.

TABLE VI.—*Size of Grade III Tumours*

| Size (cm.) | Number |
|---|---|
| 0·2–0·4 | 6 |
| 0.5–0·7 | 9 |
| 0·8–1·0 | 2 |
| over 1·0 | 2 |

TABLE VII.—*Size of Carcinomas*

| Compound | Grade I | | Grade II | | Grade III |
|---|---|---|---|---|---|
| | Small | Large | Small | Large | Large |
| Molten wax | 3 | 0 | 1 | 0 | 0 |
| Crushed wax | 2 | 0 | 0 | 0 | 0 |
| Total | 5 | 0 | 1 | 0 | 0 |
| MC (Yale) | 5 | 5 | 1 | 5 | 10 |
| MC (Leeds) | 3 | 1 | 5 | 10 | 6 |
| Total | 8 | 6 | 6 | 15 | 16 |
| Bis (2-amino-1-naphthyl) phosphate | 7 | 6 | 4 | 5 | 1 |
| 1-Amino-2-naphthol hydrochloride | 7 | 7 | 6 | 6 | 0 |
| 1-Phenylazo-2-naphthol | 7 | 2 | 0 | 1 | 0 |
| 3-Hydroxy-4-aminodiphenyl sulphate | 6 | 2 | 0 | 1 | 1 |
| 2-Naphthylhydroxylamine | 6 | 2 | 4 | 7 | 0 |
| 2-Anthramine | 3 | 4 | 2 | 1 | 1 |
| Total | 36 | 23 | 16 | 21 | 3 |

*Size of tumours.*—A division of carcinomas into small and large was made microscopically (Table VII), according to the extent of the bladder wall affected. All the tumours induced by paraffin wax alone were small; of those induced by weak carcinogens, 52 were small and 49 large; of those induced by strong carcinogens, 14 were small and 37 large.

In Table VI the size of the Grade III tumours is given. Thirteen of 19 tumours exceeded 0·5 cm. in greatest diameter, the largest being 1·3 cm. Eleven of the 13 large tumours were induced by MC. Clearly the tumours induced by strong carcinogens were larger and more invasive than those in the other two groups.

*Latent period of tumours.*—The experiments were designed so that all surviving mice were killed at 40 weeks following implantation of the pellet. At this point in time many mice in all the experiments still had no tumours and might have developed them later. When the mice dying or requiring to be killed on account of ill-health before the termination of the 40-week period are analysed (Table VIII) it is seen that the great majority (32 out of 40) of those implanted with

TABLE VIII.—*Latent Period of Tumours*

| | | Dying 0–19 weeks | | | | | Dying 20–39 weeks | | | | |
|---|---|---|---|---|---|---|---|---|---|---|---|
| | | | | Carcinoma | | | | | Carcinoma | | |
| | Total mice | No tumour | Papilloma | I | II | III | No tumour | Papilloma | I | II | III |
| Paraffin wax | 14 | — | — | — | — | — | 11 | 2 | 0 | 1 | 0 |
| Weak carcinogens | 26 | — | — | — | — | — | 21 | 1 | 4 | 2 | 0 |
| 20-Methylcholanthrene | 27 | 6 | 3 | 0 | 3 | 6 | 4 | 0 | 3 | 4 | 1 |

paraffin wax and weak carcinogens had no tumours. Of the 8 tumours which did occur only 3 were invasive. Of the mice implanted with MC, 10 out of 27 had no tumours but the striking feature is that of the 17 tumours which did occur, 7 were of Grade III. As only 16 examples of Grade III tumours occurred in the 79 bladders assessed (Table III) it can be deduced that this type of tumour is likely to be highly malignant from the start and to kill the host rapidly.

### DISCUSSION

It was claimed previously by Leeds workers that the surgical implantation of paraffin wax pellets containing suspected carcinogens into the bladders of mice was a satisfactory method of demonstrating carcinogenic potency in certain classes of compound. The present comparison of the potency of 20-methylcholanthrene relative to other carcinogens and to the vehicle alone adds considerable support to this contention. Firstly, 16 of 51 tumours induced by MC were unequivocally malignant; secondly the pre-malignant epithelial changes are those which would be expected by comparison with the action of this carcinogen in other sites, e.g. the breast or skin; and thirdly the tumours fit into a range of degree of malignancy extending from benign to highly invasive. The surprising feature is that after pellets containing even this potent carcinogen have lodged in the bladder for 40 weeks, a quarter of the organs remained normal.

*Non-neoplastic changes.*—The vehicle alone caused some degree of inflammation, epithelial hyperplasia and squamous metaplasia, but all these features were more marked when chemicals were added. The most notable feature was the high degree of squamous metaplasia induced by 1-amino-2-naphthol, a degree significantly higher than that induced by the other weak carcinogens or by MC. Bonser and Jull (1956) had noted previously that squamous metaplasia in another part of the bladder was frequently associated with the presence of a transitional cell tumour, but in the case of 1-amino-2-naphthol the high degree of squamous metaplasia was not associated with as high an incidence of tumours as that caused by MC. The metaplasia seems to have been a specific effect of the carcinogen.

*Neoplastic changes.*—In assessing the incidence of tumour types, where they were multiple each individual tumour was counted. Thus the total incidence is higher than in previous publications, where individual mice were counted. The incidence of papillomas increased from 3 per cent in bladders implanted with wax to 9 and 19 per cent in those implanted with weak and strong carcinogens respectively. As papillomas can be seen to be undergoing malignant changes, this rising incidence is to be expected.

When the carcinomas are considered, not only is there a rising incidence from vehicle to strong carcinogen but the grade, size and invasive properties are greatly increased when MC is the carcinogen (Tables IV, V, VI and VII). In addition, the latent period is reduced and tumours highly malignant from the start occur before 20 weeks following the implantation of MC (Table VIII). On microscopical examination one-third of the malignant tumours induced by MC were of the highly invasive Grade III, and five were of squamous type, the only tumours of this type to occur in the whole experiment. Tumours in other sites (e.g. breast and uterus) induced by this carcinogen are known to have a similar structure.

The use of 20-methylcholanthrene in a well-controlled experiment has thus added considerable support to the contention that the surgical implantation of wax pellets containing a carcinogen is a useful method for testing substances for which other methods of testing are not suitable. None of the metabolites of the aromatic amines approached MC in carcinogenic potency.

### SUMMARY

Surgical implantation into the bladder of the mouse of paraffin wax pellets containing suspected carcinogens such as aromatic amines and their metabolites has been used as a method for detecting carcinogenic activity in Leeds for many years. The yield of malignant tumours was low, not exceeding 32 per cent, when the experiments were terminated at 40 weeks.

The method was therefore further investigated by using 20-methyl-cholanthrene as a " strong " carcinogen and the yield of malignant tumours was raised to 54 per cent.

Non-neoplastic changes were of a similar order whether weak or strong carcinogens were used, except for an unusually high incidence of squamous metaplasia when 1-amino-2-naphthol was the carcinogen.

Papillomas and invasive carcinomas were more abundant when 20-methylcholanthrene was the carcinogen. Of 37 invasive carcinomas of the latter series, 16 had invaded another organ, such as peritoneal adipose tissue, vagina, uterus, breast, etc. (Table V). The size of invasive MC tumours was greater and the latent period was less.

These results show that the method is a satisfactory one for detecting and assessing the carcinogenic activity of suspected carcinogens.

### REFERENCES

Allen, M. J., Boyland, E., Dukes, C. E., Horning, E. S. and Watson, J. G.—(1957) *Brit. J. Cancer*, **11**, 212.

Bonser, G. M., Boyland, E., Busby, E. R., Clayson, D. B., Grover, P. L. and Jull, J. W.—(1963) *Ibid.*, **17**, 127.

*Idem*, Bradshaw, L., Clayson, D. B. and Jull, J. W.—(1956) *Ibid.*: **10**, 539.

*Idem* and Jull, J. W.—(1956) *J. Path. Bact.*, **72**, 489.

Clayson, D. B., Jull, J. W. and Bonser, G. M.—(1958) *Brit. J. Cancer*, **12**, 222.

Jull, J. W.—(1951) *Ibid.*, **5**, 328.